# New insights on an old friend: AroA linked to iron-dependent outer membrane stability

**DOI:** 10.1128/mbio.02799-24

**Published:** 2024-11-21

**Authors:** Priscilla Chin, Christopher J. Anderson

**Affiliations:** 1 Centre for Inflammation Research, Institute for Regeneration and Repair, University of Edinburgh, Edinburgh, United Kingdom; Yale School of Medicine, New Haven, Connecticut, USA

**Keywords:** *Salmonella*, iron utilization, outer membrane

## Abstract

*Salmonella* is a common causative agent of infectious intestinal and systemic disease and has been extensively studied for several decades. Yet, much of *Salmonella* pathogenicity remains a mystery due in part to the highly complex virulence and adaptation strategies at the pathogen’s disposal. One of the more influential tools within the field, an attenuated *aroA-*deficient *Salmonella* strain, has been used for many years to probe the host immune response that would otherwise be impossible with a fully virulent strain. Now, new work by Rooke et al. (J. L. Rooke, E. C. A. Goodall, K. Pullela, R. Da Costa, et al., mBio 15:e03319-23, 2024, https://doi.org/10.1128/mbio.03319-23) utilizes in-depth transposon-directed insertion-site sequencing to elucidate the contribution of genes to *Salmonella* fitness within isogenic wild-type and *aroA*-deficient strains. Specifically, Rooke et al. demonstrate that the deletion of the *aroA* gene leads to iron-dependent membrane instability, raising several exciting new ideas surrounding *Salmonella* biology and therapeutic strategies.

## COMMENTARY

*Salmonella enterica* infection causes gastroenteritis in humans through the ingestion of contaminated food and water and, depending on the strain and the host, systemic infections with a high mortality rate. The bacteria can be designated as one of >2,500 serovars on the basis of genetic content and ability to infect or colonize different host species (1). In addition to humans, one of the most common serovars, serovar Typhimurium, can also infect animal hosts and is thus widely studied. Given the global prevalence and burden as well as the emergence of antibiotic resistance, there remains an urgent need for the development of vaccines and/or new alternatives to antimicrobial therapies.

Two of the most well-characterized and impactful *Salmonella* virulence mechanisms are the two type-3 secretion systems (T3SS) encoded within *Salmonella* pathogenicity islands 1 and 2 (SPI-1 and -2). Generally speaking, *Salmonella* utilizes the SPI-1-encoded T3SS to invade non-phagocytic intestinal epithelial cells and generate intestinal inflammation (2, 3). The internalized *Salmonella* is then enclosed within vesicles, known as *Salmonella*-containing vacuoles (SCVs), that permit persistent survival and replication in the host cell. The T3SS encoded within SPI-2 supports bacterial localization and replication within the SCV of epithelial cells and phagocytes (4), and intracellular survival subsequently promotes the dissemination and circulation throughout the lymphatic system to colonize systemic tissue (1, 5). These critical virulence factors lead to a fatal infection in laboratory mice (6), which has been linked, at least in part, to a mutant allele of the host solute carrier Slc11a1 (previously referred to as Nramp) (7, 8). A significant portion of *Salmonella* research has focused on the immunogenicity and host response to systemic infection by frequently utilizing attenuated *Salmonella* strains due to the widespread use of the highly susceptible C57BL/6 (Slc11a1 mutant) background in mouse genetics (9). Often, avirulent *Salmonella* strains are used as a live vaccine candidate to confer protection against virulent challenge (9–12).

Almost 50 years ago, Hoiseth and Stocker (10) developed an aromatic amino acid auxotroph *S*. Typhimurium strain (named SL3261) by deleting the *aroA* gene from the parental strain *S*. Typhimurium SL1344 as part of a transposon-directed mutagenesis screen. SL3261, as well as independently constructed Δ*aroA* mutants, is effective as a live oral vaccine in mice as infection with this mutant subsequently confers protection once challenged with pathogenic *Salmonella* (9, 13, 14). Since AroA forms part of the shikimate pathway to link glycolysis to aromatic amino acid production via chorismate synthesis (15), SL3261 is unable to synthesize aromatic amino acids required for growth, corresponding with the original identification and selection of this mutant (10). Despite being widely used for translational purposes, such as vaccines and anticancer therapeutics, there is a lack of understanding of the molecular mechanism for the attenuation of SL3261 and related *aroA* deletion strains (9–11). The widely accepted model that has permeated the field is that *aroA*-deficient strains are attenuated for intracellular growth and survival during systemic infection because of their inability to create endogenous aromatic amino acids, thereby inferring that the intracellular host environment (SCV) is a poor source of exogenously provided aromatic amino acids.

In a recent study, Rooke et al. (16) used transposon-directed insertion-site sequencing (TraDIS) to generate and sequence transposon mutant libraries in both virulent *S*. Typhimurium SL1344 and non-virulent SL3261 *in vitro*. TraDIS allows for large-scale genome-wide genotype-phenotype relationship analysis of bacteria where many mutants can be screened in a timely manner to determine gene essentiality, function, and genetic interactions. The detection of transposon insertion sites is directly correlated with the fitness of the corresponding mutant and can also be compared to published databases as a benchmark (17). Numerous new gene functions in different bacterial species have been identified using this powerful technique, and the high resolution of TraDIS allows for the comparison of two very closely related *Salmonella* strains (isogenic SL1344 and SL3261) (18). Rooke et al. searched for transposon insertions that were statistically underrepresented in their sequencing data, as this implies that these genes are essential or carry a high fitness cost if mutated/deleted. With their clever study design, Rooke et al. were able to identify essential/high fitness cost genes that were conserved across published transposon mutant libraries in multiple strains of *Salmonella* (core genes), strain (SL1344)-specific essential genes, and genes unique to the *aroA*-deficient SL3261, which would then provide clues for the functional pathways linked to AroA-dependent attenuation.

As expected, and in line with the existing dogma surrounding the importance of AroA in the synthesis of aromatic amino acids, the authors discovered significant differences in genes involved in aromatic amino acid import with less abundant transposon insertions within the tyrosine transporter *tyrP* in the *aroA*-deficient SL3261 strain. Additionally, insertional mutations in the ferrous iron (Fe^2+^) transport system *feoABC* were also significantly underrepresented in the *aroA*-deficient SL3261, suggesting a requirement of ferrous iron for this strain to grow. Indeed, AroA-dependent chorismate synthesis is a required precursor for siderophore production. These proteins play a major role in scavenging ferric iron (Fe^3+^) from the environment, suggesting a forced dependence on ferrous iron in the context of *aroA* deficiency. While wild-type SL1344 had a lower abundance of mutants in the ferric iron transporters (*fepBCDG*) and its growth in iron-limiting conditions was rescued by supplementation with ferric iron, the *aroA*-deficient strain was more sensitive to iron-limiting growth conditions and insensitive to ferric iron supplementation. Of note, Rooke et al. uncovered that the historical SL3261 strain was a double mutant lacking *aroA* and having an in-frame truncation of the periplasmic metalloprotease encoding *ycaL*. However, the complementation of SL3261 with *aroA* restored the ability of this strain to cope with iron limitation.

Surprisingly, transposon insertions for multiple genes associated with outer membrane biosynthesis were differentially abundant between wild-type and *aroA*-deficient strains. Many of these same genes were previously identified as being linked to iron restriction, suggesting a potential link between AroA, membrane permeability, and iron utilization (19). To test this, Rooke et al. treated wild-type or *aroA*-deficient SL3261 with membrane-acting compounds (vancomycin, bile salts, and EDTA) with or without siderophore (enterobactin) supplementation. The authors found that SL3261, as well as the *aroA* single-deletion strain, were more sensitive to membrane-acting compounds in an AroA and siderophore-dependent manner. Membrane defects in Δ*aroA* were previously reported with partial explanations of this phenomenon offered including defects in ubiquinone and peptidoglycan synthesis (13, 20). Now, a third potential explanation has been added to the mix where the restricted ferric iron uptake in the absence of AroA leads to increased membrane permeability.

These findings raise several interesting questions that merit further exploration, especially considering how AroA-deficient strains are widely used for studying host immune responses. In particular, does iron-dependent membrane permeability explain intracellular and systemic attenuation of *aroA* mutant strains? Furthermore, is the immunological protection provided by *aroA* mutants against subsequent infection simply in relation to the strain being less adept at survival or is this more directly related to changes in bacterial membrane permeability? Iron is crucial for *Salmonella* evasion of the immune system and colonization (21, 22), and Slc11a1 has been shown to limit intracellular iron concentrations for *Salmonella* (23). Therefore, it is tempting to speculate that AroA-dependent fitness would be linked to host Slc11a1-dependent iron restriction within the SCV. Recent studies have revealed iron-independent functions for Slc11a1 (24, 25), so this hypothesis requires testing. Future investigations are necessary to interrogate the AroA-dependent membrane defects *in vivo* and within the various tissue and cellular microenvironments that *Salmonella* encounters during infection. Insights into this area might not only provide clarity on a long-used tool within the field but also reveal novel *Salmonella* biology and potential targets for therapeutic and/or vaccine-based manipulations.
